# Assessing the Efficacy of *Bacillus thuringiensis* (Bt) Pyramided Proteins Cry1F, Cry1A.105, Cry2Ab2, and Vip3Aa20 Expressed in Bt Maize Against Lepidopteran Pests in Brazil

**DOI:** 10.1093/jee/toy380

**Published:** 2018-12-17

**Authors:** Luiz H Marques, Antonio C Santos, Boris A Castro, Valeria F Moscardini, Jaedino Rosseto, Oscar A B N Silva, Jonathan M Babcock

**Affiliations:** 1 Dow AgroSciences Industrial Ltda, Av. Nações Unidas, São Paulo, Brazil; 2 Dow AgroSciences LLC, Indianapolis, IN

**Keywords:** *Helicoverpa zea*, Diatraea saccharalis, Elasmopalpus lignosellus, Agrotis ipsilon, Bt maize

## Abstract

Field studies across four states in maize-producing areas of Brazil were conducted to characterize the efficacy of a new pyramided *Bacillus thuringiensis* (Bt) Berliner technology in maize, *Zea mays* L., and compare it to existing single and pyramided commercial Bt technologies, to control *Helicoverpa zea* Boddie (Lepidoptera: Noctuidae), *Elasmopalpus lignosellus* Zeller (Lepidoptera: Pyralidae), *Agrotis ipsilon* (Hufnagel) (Lepidoptera: Noctuidae), and *Diatraea saccharalis* F. (Lepidoptera: Crambidae). Bt maize expressing Vip3Aa20 protein and pyramided Bt maize expressing proteins Cry1F + Cry1A.105 + Cry2Ab2 + Vip3Aa20 provided excellent protection against kernel feeding by *H. zea* compared to Bt technologies expressing only Cry1F or Cry1A.105 + Cry2Ab2. Bt maize expressing Cry1F, Cry1A.105 + Cry2Ab2, Cry1F + Cry1A.105 + Cry2Ab2, and Cry1F + Cry1A.105 + Cry2Ab2 + Vip3Aa20 resulted in less than 5% of plants injured by *E. lignosellus*, significantly less than Bt maize expressing only Vip3Aa20 and a non-Bt maize iso-hybrid with and without a thiamethoxam seed treatment. The highest protection against plant cutting injury caused by *A. ipsilon* was observed in the pyramid Bt maize technology expressing Cry1F + Cry1A.105 + Cry2Ab2 + Vip3Aa20. However, it did not differ statistically from the Bt maize expressing Vip3Aa20, Cry1F, or Cry1F + Cry1A.105 + Cry2Ab2. All Bt maize hybrids evaluated in our study were highly effective in reducing tunneling injury caused by *D. saccharalis*. These results show that a new maize technology expressing pyramided Bt proteins Cry1F + Cry1A.105 + Cry2Ab2 + Vip3Aa20 offers a higher level of protection from feeding by the above lepidopteran pest complex compared to maize with a single Bt protein or double pyramided Bt proteins.

Brazil is one of the largest agricultural producers in the world, a status facilitated by scientific and technical advances that modernized its agriculture over the past 40 yrs (de Souza et al. 2013). The adoption of agricultural biotechnology in Brazil during the past decade played an important role for the increase in productivity, helping Brazil reach a position among the three largest agricultural producers in the world (Céleres 2012, de Souza et al. 2013). Brazil planted 49.1 million hectares of biotechnology-derived varieties of maize, *Zea mays* L., soybean, *Glycine max* (L.) Merrill, and cotton, *Gossypium hirsutum* L., in the 2016/2017 growing season. It represented 93.4% of the total area of 52.5 million hectares planted in Brazil to these three crops (Céleres 2017) and 27% of the world’s 185.1 million hectares of biotech crop production (ISAAA 2016). Brazil is currently second in the world in area planted to biotech crops, following the United States with 72.9 million hectares, or 39% of the global total, and followed by Argentina with 23.8 million hectares, or 13% of the total global biotech crop production (ISAAA 2016). The economic benefit for Brazil is estimated at US$16.4 billion since biotechnology was adopted (ISAAA 2016). Brazil was relatively a late adopter of biotech crops (de Souza et al. 2013). Maize hybrids engineered to express insecticidal proteins derived from the soil bacterium *Bacillus thuringiensis* (Bt) were first approved in Brazil in 2007 (ISAAA 2018) with the registration of event MON810, expressing the Cry1Ab delta-endotoxin. Since then, Brazil has approved 44 biotech events (single or pyramided) in maize alone (ISAAA 2018). They express single, stacked and pyramided proteins including Cry1F approved in 2008 (CTNBio 2008), Cry1A.105 and Cry2Ab2 in 2009, VIP3Aa20 in 2009, etc. (de Souza et al. 2013, ISAAA 2018). Biotech maize plantings in Brazil reached 15.7 million hectares in the 2016/2017 growing season, 5.3 million in summer planting (safra) and 10.4 million in winter planting (safrinha). Its total planting represented 88.4% adoption rate of biotech maize, including 63.9% stacked with insect resistance/herbicide tolerance, 20.7% with insect resistance alone, and 3.8% with herbicide tolerance alone (Céleres 2017).

Bt insecticidal proteins Cry1Ab, Cry1F, Cry1A.105, Cry2Ab2, and Vip3Aa20, were bio-engineered into maize plants (Bt maize) to protect against a broad range of lepidopteran pests (ISAAA 2018) that feed on aerial plant parts including stalk, foliage and ear. The Cry1F insecticidal protein is a δ-endotoxin derived from *B. thuringiensis* var. *aizawai* expressed in maize transgenic event TC1507 (Storer et al. 2012a). Cry2Ab2, derived from *B. thuringiensis* subsp. *kumamotoensis,* and Cry1A.105, a synthetic chimera of Cry1Ab, Cry1Ac, and Cry1F, are δ-endotoxins expressed in transgenic event MON 89034 (EPA 2010, Carrière et al. 2016a, ISAAA 2018). Vip3Aa20, derived from *B. thuringiensis* strain AB88 (Estruch et al. 1996), is expressed in maize transgenic event MIR162. Insecticidal Bt proteins Cry1F, Cry2Ab2, and Cry1A.105 belong to the crystal (Cry) protein family, whereas Vip3Aa20 belongs to the vegetative insecticide protein (VIP) family (Estruch et al. 1996, Carrière et al. 2016a). These insecticidal proteins interact with specific receptors in the insect gut and induce the formation of pores in the apical membrane of the cells, destroying the intestinal tissue of target insects, resulting in larval death (Raymond et al. 2010, Gómez et al. 2014).

Tropical conditions in Brazil often result in continuous infestation exposure to a broad range of lepidopteran pests during the entire maize crop development cycle. Secondary pests such as *Agrotis ipsilon* (Hufnagel) (Lepidoptera: Noctuidae) and *Elasmopalpus lignosellus* Zeller (Lepidoptera: Pyralidae) affect the maize crop during seedling and early developmental stages. *Diatraea saccharalis* F. (Lepidoptera: Crambidae) is also considered a secondary pest in Brazil and affects the vegetative and reproductive crop stages. Injury levels caused by these secondary pests vary by region, but may become of key importance in some areas of Brazil. *Helicoverpa zea* Boddie (Lepidoptera: Noctuidae) is an economically important lepidopteran pest of maize across Brazil. Its main injury is caused during maize reproductive ear-development stages, where major losses often result from direct feeding on developing kernels (Valicente 2015).

Lepidopteran pest management in Brazil historically relied on the use of chemical insecticides. However, many important pests of maize have cryptic feeding habits (e.g., inside plant parts) or seek hiding places (soil debris), making it difficult to monitor or to reach with chemical insecticides alone. Therefore, current pest control in Brazilian maize is the result of a broad adoption of integrated practices (Piccinin et al. 2016). The success of these programs led to current pest management practices in maize relying on integrating multiple strategies, including cultural, biological, chemical and transgenic methods (Okumura et al. 2013, Waquil et al. 2013, Piccinin et al. 2016, Burtet et al. 2017). As part of this integrated strategy, Bt crops offer a practical management of lepidopteran target pests. It is compatible with other control methods and, therefore, contributes to improved yields, reduced use of conventional insecticide applications, reduced labor costs, lower environmental impact, less fungal contamination of grain and a low risk to non-target organisms (Huesing and English 2004, Naranjo 2009, Lu et al. 2012, Dively et al. 2018). However, the development of insect field resistance to Bt traits can undermine the efficacy of Bt crops and threatens the sustainability of the environmental and economic benefits of this technology (Farias et al. 2014, Santos-Amaya et al. 2015). The Cry1F protein was one of the most effective Bt transgenic tools to control *Spodoptera frugiperda* (J. E. Smith) (Lepidoptera: Noctuidae) in Brazil when it was made commercially available in 2009. However, reduced field efficacy was reported in some areas in Brazil in 2011 and field-evolved resistance of *S. frugiperda* to Cry1F was confirmed in 2014 (Farias et al. 2014).

Transgenic crop ‘pyramids’, which include two or multiple modes of action against the same pest (Storer et al. 2012b), were developed to improve efficacy, broaden the spectrum of pests controlled and delay the evolution of resistance in target insect pest populations (Carrière et al. 2015, 2016a, b; Santos-Amaya et al. 2015). The combined effect of multiple Bt toxins can improve protection from damage caused by a broader range of pests, improve the efficacy against individual pest species and reduce pest injury compared to Bt crops with a single mode of action (Adamczyk Jr. and Gore 2004, Burkness et al. 2010, Siebert et al. 2012). Seed technology developers introduced commercial maize hybrids with pyramided transgenes in 2008 (Onstad et al. 2011). Bt pyramids are now commercially available in different countries. The United States and Australia have totally replaced single-toxin Bt cotton with pyramided Bt cotton technologies. Pyramided Bt crops are expected to become more dominant in the future, because they can enhance resistance management as well as pest control (Brévault et al. 2013).

Dow AgroSciences developed a Bt maize technology with pyramided transgenic events TC1507 × MON 89034 × MIR162 × NK603 through conventional breeding of lines containing the single events. This Bt transgenic pyramid received approval for commercial cultivation in Brazil in 2017 (CTNBio 2017, ISAAA 2018). This pyramid of transgenic events expresses the proteins Cry1F and PAT (event TC1507), Cry1A.105 and Cry2Ab2 (event MON 89034), Vip3Aa20 (event MIR162), and CP4 EPSPS (event NK603). Multiple modes of action are expressed against a broad range of lepidopteran pests through the expression of the proteins Cry1F, Cry1A.105, Cry2Ab2 and Vip3Aa20, and dual tolerance to glyphosate and glufosinate herbicides through the proteins CP4 EPSPS (5-enolpyruvulshikimate-3-phosphate synthase enzyme) and PAT (phosphinothricin N-acetyltransferase enzyme), respectively. During the early stage of development, inbred lines were introgressed with events TC1507, MON 89034, MIR162, and NK603 through classical breeding to be the parents of the pyramided commercial hybrids.

No previous publication exists on the combined target pest efficacy of transgenic events TC1507 × MON 89034 × MIR162 in Bt maize. Therefore, an objective of these experiments were to evaluate the field efficacy of pyramided Bt maize expressing Cry1F + Cry1A.105 + Cry2Ab2 + Vip3Aa20 proteins (events TC1507 × MON 89034 × MIR162) to control *H. zea, E. lignosellus, A. ipsilon*, and *D. saccharalis* in Brazil. A second objective was to compare the efficacy of the Bt pyramid to those of Bt maize technologies containing individual parental transgenic events and to a seed treatment (against *E. lignosellus*) across several maize planting regions in Brazil.

## Materials and Methods

### Site Locations and Experimental Design

Field experiments were conducted from 2015 to 2017. Four Brazilian states were selected for the experiments, representing important commercial maize growing regions and including a broad range of climatic and agronomic conditions normally observed in maize growing areas of Brazil (Table 1). Since the research was conducted prior to commercial approval of TC1507 × MON 89034 × MIR162 maize, all research was conducted following strict adherence to Brazilian field trial permit requirements at accredited certified field research sites, which included Dow AgroSciences, and SGS field research stations. All field experiments followed a randomized complete block design (RCBD) with four replications. Plot size varied among locations from five (5.0) to six (6.0) m in length and seven or eight rows wide. Row spacing in all locations was 50 cms. Field plots received no foliar insecticide applications. Artificial irrigation (overhead sprinkler) was available at all sites and occasionally was used as needed to avoid water stress during times of drought. Commercial herbicides and fungicides were applied following local weed and disease control management practices. Seeds were planted with a manual seed planter or precision air-planter at the rate of ~70,000 seeds per ha. Tillage practices implemented included no tillage (Cascavel/Palotina, PR and Rio Verde/Montividiu, GO), reduced tillage (Indianópolis, MG), and conventional tillage (Conchal/Mogi Mirim, SP).

**Table 1.** Field location, planting time, artificially infested pest and data type collected from trials in Brazil, 2015 to 2017

Location (city, state^*a*^)	Planting time (month, year)	Pest (instar infested^*b*^)	Data type collected
Indianópolis, MG	Mar., 2015	*H. zea* (L1)*, A. ipsilon* (L3)	Kernel consumed area, percent of cut plants
Conchal, SP	April, 2015	*H. zea* (L1)*, A. ipsilon* (L3)	Kernel consumed area, percent of cut plants
Palotina, PR	Mar., 2015	*A. ipsilon* (L3)	Percent of cut plants
Indianópolis, MG	Jan., 2016	*H. zea* (L1), *A. ipsilon* (L3)*, E. lignosellus* (L3)	Kernel consumed area, percent of cut plants, percent of dead or injured plants
Indianópolis, MG	Nov., 2016	*H. zea* (L1)*, A. ipsilon* (L3)*, D. saccharalis* (L1)	Kernel consumed area, percent of cut plants, tunnel length
Indianópolis, MG	Nov., 2016	*H. zea* (L1)*, E. lignosellus* (L3)*, D. saccharalis* (L1)	Kernel consumed area, percent of dead or injured plants, tunnel length
Indianópolis, MG	Nov., 2016	*H. zea* (L3)*, A. ipsilon* (L3)*, D. saccharalis* (L1)	Kernel consumed area, percent of cut plants, tunnel length
Indianópolis, MG	Nov., 2016	*H. zea* (L3)*, E. lignosellus* (L3)*, D. saccharalis* (L1)	Kernel consumed area, percent of dead or injured plants, tunnel length
Indianópolis, MG	Dec., 2016	*H. zea* (L3)*, A. ipsilon* (L3)*, D. saccharalis* (L1)	Kernel consumed area, percent of cut plants, tunnel length
Indianópolis, MG	Dec., 2016	*H. zea* (L1)*, E. lignosellus* (L3)	Kernel consumed area, percent of dead or injured plants
Rio Verde, GO	Jan., 2016	*H. zea* (L1)*, A. ipsilon* (L3)	Kernel consumed area, percent of cut plants
Rio Verde, GO	Nov., 2016	*H. zea* (L1)*, A. ipsilon* (L3)*, E. lignosellus* (L3)*, D. saccharalis* (L1)	Kernel consumed area, percent of cut plants, percent of dead or injured plants, tunnel length
Palotina, PR	Jan., 2016	*H. zea* (L1)*, A. ipsilon* (L3)*, E. lignosellus* (L3)	Kernel consumed area, percent of cut plants, percent of dead or injured plants
Mogi Mirim, SP	Mar., 2017	*A. ipsilon* (L3)	Percent of cut plants
Rio Verde, GO	Mar., 2017	*A. ipsilon* (L3)	Percent of cut plants
Rio Verde, GO	Jan., 2017	*H. zea* (L1)*, A. ipsilon* (L3)	Kernel consumed area, percent of cut plants
Rio Verde, GO	Jan., 2017	*H. zea* (L1)*, E. lignosellus* (L3)	Kernel consumed area, percent of dead or injured plants
Rio Verde, GO	Mar., 2017	*H. zea* (L1)	Kernel consumed area
Cascavel, PR	Jan., 2017	*A. ipsilon* (L3)	Percent of cut plants
Cascavel, PR	Jan., 2017	*H. zea* (L1)*, E. lignosellus* (L3)	Kernel consumed area, percent of dead or injured plants
Cascavel, PR	Mar., 2017	*A. ipsilon* (L3)	Percent of cut plants
Cascavel, PR	Mar., 2017	*A. ipsilon* (L3)*, E. lignosellus* (L3)	Percent of cut plants, percent of dead or injured plants

^*a*^Brazilian states for field trials: MG = Minas Gerais; SP = São Paulo; PR = Paraná and GO = Goiás.

^*b*^Instar infested: L1 = first instar, L3 = third instar.

### Treatments

Treatments consisted of experimental (noncommercial) maize hybrids developed by Dow AgroSciences LLC (Indianapolis, IN). All hybrids contained the same genetic background across all years and locations and expressed different Bt events depending on the treatment. A non-Bt isogenic maize hybrid (iso-hybrid) of the same genetic background was used as control (Table 2). All seeds selected for planting passed quality assessment checks testing for gene expression, adventitious presence, germination, etc. During field evaluations, plants in Bt plots showing lepidopteran injury were checked with test strips (QuickStix Strips, EnviroLogix Inc., Portland, ME) to exclude possible injury data from non-Bt plants inside Bt plots. Non-Bt control plots did not receive insecticide applications with the exception of field experiments against *E. lignosellus*. In this case, field trials contained two non-Bt controls (Table 2). The second non-Bt control included an insecticidal seed treatment with thiamethoxam applied at 42 g a.i./ 60.000 seeds (Cruiser 350 FS, Syngenta, Basel, Switzerland).

**Table 2.** Treatments (maize hybrids with the expressed Bt proteins if applicable), and corresponding Bt events

Treatments	Event (s)
Cry1F	TC1507^*a*^
Vip3Aa20	MIR162^*b*^
Cry1A.105 + Cry2Ab2	MON 89034^*c*^
Cry1F + Cry1A.105 + Cry2Ab2	TC1507 × MON 89034 × NK603^*d*^
Cry1F + Cry1A.105 + Cry2Ab2 + Vip3Aa20	TC1507 × MON 89034 × NK603 × MIR162
Non-Bt maize Iso-hybrid + Seed treatment (Thiamethoxam at 42 g a.i./ 60.000 seeds)	None
Non-Bt maize Iso-hybrid	None

^*a*^Event TC1507 expresses Cry1F and PAT proteins. PAT protein confers glufosinate herbicide tolerance, Dow AgroSciences, Indianapolis, IN.

^*b*^Event MIR162 expresses Vip3Aa20 protein, Syngenta, Research Triangle Park, NC.

^*c*^Event MON 89034 expresses Cry1A.105 + Cry2Ab2 proteins, Monsanto Company, St. Louis, MO.

^*d*^Event NK603 expresses CP4EPSPS protein that confers glyphosate herbicide tolerance, Monsanto Company, St. Louis, MO.

### Insect Pest Source

All treatments were evaluated against *H. zea, E. lignosellus, A. ipsilon*, and *D. saccharalis* using artificial infestations in all locations to ensure uniform pest pressure across experimental plots. Insects were obtained from laboratory colonies maintained by Dow AgroSciences (Mogi Mirim Research Center, Mogi Mirim – São Paulo state) or Bug Agentes Biológicos (Charqueada – São Paulo state). All insect colonies were reared on artificial diet and maintained in a room with controlled conditions of temperature (25 ± 3°C), relative humidity (60 ± 5%) and photoperiod (14:10 (L:D) h). Pest population colonies were rejuvenated every year with insects from non-Bt soybean or non-Bt maize fields, except for *D. saccharalis*, which were collected from non-Bt sugarcane fields. Injury from natural infestations of these pest species was not detected or recorded from evaluated plants in any of the field trials.

### Insect Infestation Procedures

#### Helicoverpa zea

Ten plants per plot were randomly selected from one of the middle rows. Selected plants were tagged and artificially infested at the R1 (Ritchie et al. 1982) maize growth stage. Most field trials were infested with five first instars. Three trials in Indianópolis (MG) were infested with five third instars (Table 1), due to lack of availability of first instars. Larvae were placed on the primary ear of each selected plant using a soft camel’s hair brush. Kernel-feeding injury was assessed at the R3 maize growth stage by measuring the total area (cm^2^) of *H. zea*-attributed feeding to the primary ear kernels.

#### Diatraea saccharalis

Ten plants per plot were selected randomly from one of the two middle rows. Selected plants were tagged and artificially infested. The first infestation was performed at the V7 stage and a second infestation conducted at the VT corn growth stage (Ritchie et al. 1982). *Diatraea saccharalis* neonate larvae (L1 stage) were mixed with corn cob grits and then transferred into plastic dispensers commonly referred to as bazookas (Davis and Oswalt 1979). Each bazooka shot was calibrated to deliver 10 first instars. One bazooka shot was delivered per plant. Therefore, a total of 20 larvae were infested per plant, 10 larvae at V7 and 10 larvae at VT stage. Larvae were deposited in the whorl at V7 and in the axil of the leaf closest to the primary ear during the VT stage. Plant injury was assessed 21 days after the second infestation (DAI) by dissecting plant stalks and measuring the length of plant tunneling (cm) resulting from insect feeding. The percent injured plants for each plot was calculated based on the number of plants displaying tunnel injury versus the total number of infested plants.

#### Elasmopalpus lignosellus

Twenty healthy plants per plot were selected from one of the center rows of each plot. A protected infestation arena was prepared to prevent insect escape by surrounding each selected plant with a cross-cut polyvinyl chloride (PVC) pipe (15 cm diameter and 12 cm height) placed at soil level. When maize plants emerged and reached the VE growth stage (Ritchie et al. 1982), two third instars per plant were deposited and confined within each individual PVC pipe arena. Larvae were transferred from diet cups to Eppendorf tubes (one larva per tube). Two open Eppendorf tubes were positioned near the base of the plant inside the PVC pipe arena, allowing the larvae to exit the tubes and infest the plant in a no-choice situation. Plant injury was assessed 21 DAI by counting the number of plants displaying dead-heart symptoms and abnormal tillering behavior resulting from insect feeding. These two symptoms were together considered as plant mortality. The percent plant mortality for each plot was estimated based on the number plants displaying dead heart and tillering symptoms versus the total number of infested plants.

#### Agrotis ipsilon

A block of 20 continuous healthy plants at seedling stage per plot was selected and artificially infested at the VE maize growth stage (Ritchie et al. 1982) by placing one third instar at the base of each plant using a pair of soft forceps. Larvae were confined to the area of the seedling plants by placing barriers made of polyethylene plastic or galvanized iron (27 cm in height, 1.5 m length and 80 cm width) around each block of selected plants to prevent insect escape. Plant injury was assessed 15 DAI by counting the number of plants exhibiting insect feeding injury, which normally occurs at soil level. Percent plant mortality in each plot was estimated based on the number of severed (cut) plants at soil level versus the total number of infested plants.

### Statistical Analyses

Data from ear-feeding injury caused by *H. zea* and the percentage of plant mortality caused by *A. ipsilon* and *E. lignosellus* were separately subjected to a linear mixed model adjusted for a RCBD in which treatments were not identically the same (PROC MIXED). These analyses were followed by a Tukey’s test to separate treatment means. Treatment was considered a fixed effect while trial, block, and the interaction treatment × trial were considered random effects. Prior to the combined (across-trial) analyses, each trial was individually analyzed and the mean squared error of the residue (MSE) was used to evaluate the homogeneity of the variance error. Data from these trials were analyzed together (pooled) as the quotient between the largest and smallest MSE was ≤7, indicating the trials were homogeneous (Pimentel-Gomes 2009). Ear-feeding injury and plant mortality percentages were transformed using either square root [√(x+0.5)] or arcsine [√(x/100)] to improve the variance assumption of normal distribution (PROC UNIVARIATE). Non-transformed data are presented in all figures. Data on proportion of plants injured by *D. saccharalis* were analyzed using Chi-square (PROC FREQ). All analyses were performed using SAS System 9.0 (SAS Institute 2008) with α = 0.05.

## Results

**Fig. 1.**
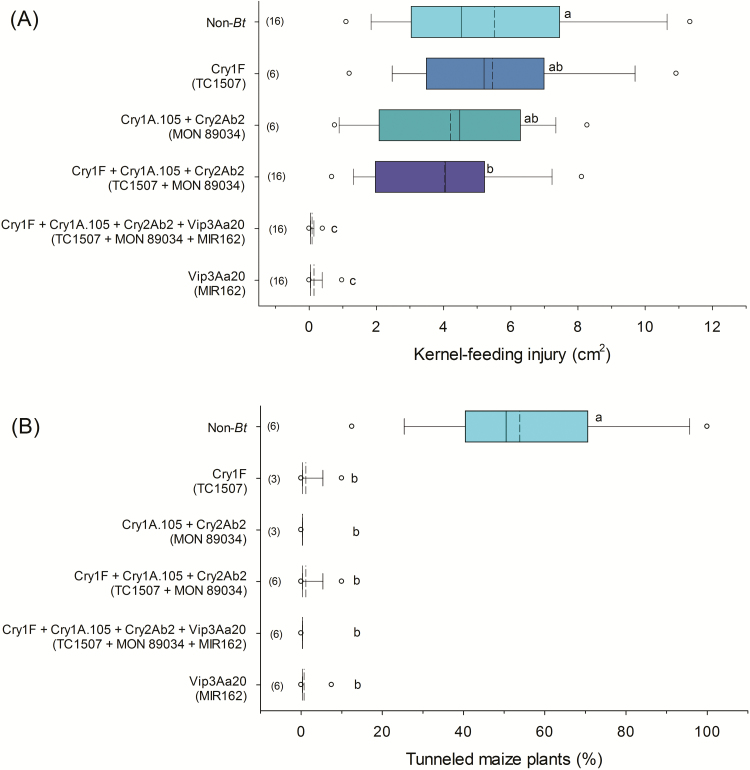
Feeding injury caused by (A) *Helicoverpa zea* and (B) *Diatraea saccharalis* on Bt and non-Bt maize plants under artificial field infestations. Number of trials are indicated within parentheses. The dashed line within a box represents the mean. The solid line within a box represents the median. The ‘o’ in the boxplots represents 95th percentiles. Boxplots of *H. zea* and *D. saccharalis* followed by different letters were significantly different by Tukey’s or Chi-square tests (α = 0.05), respectively. The vertical axis lists protein names and, in parentheses, the names of the transgenic events.

The single event maize hybrid with Vip3Aa20 (event MIR162) and the pyramid maize hybrid with Cry1F + Cry1A.105 + Cry2Ab2 + Vip3Aa20 proteins (events TC1507 × MON 89034 × MIR162), significantly reduced ear-feeding-injury caused by *H. zea* compared to the non-Bt iso-hybrid treatment and to all other treatments (F_5, 57.6_ = 72.66; *P* < 0.0001). The mean amount of kernel feeding in these two treatments was 0.1 ± 0.0 cm^2^, a reduction in ear-feeding injury by *H. zea* of close to 100% compared with the non-Bt iso-hybrid treatment (mean= 5.5 ± 0.4 cm^2^). Injury observed in technologies expressing Cry1F (TC1507) and Cry1A.105 + Cry2Ab2 (MON 89034) proteins, did not differ significantly from that of the non-Bt control treatment. Pyramided Bt maize with Cry1F + Cry1A.105 + Cry2Ab2 proteins (TC1507 × MON89034) did not significantly reduce kernel-feeding injury caused by *H. zea* compared to the single events, but the feeding injury observed in this technology (3.9 ± 0.3 cm^2^) was approximately 27% lower and statistically different compared to the non-Bt iso-hybrid (Fig. 1A).

All Bt maize technologies evaluated significantly reduced the percent of plants tunneled by *D. saccharalis* compared to the non-Bt iso-hybrid treatment (χ^2^ = 348.5; df = 6; *P* < 0.001). An average of 53.3% of non-Bt iso-hybrid plants were injured, and had a mean tunnel length of 8.1 ± 1.7 cm (Fig. 1B).

**Fig. 2.**
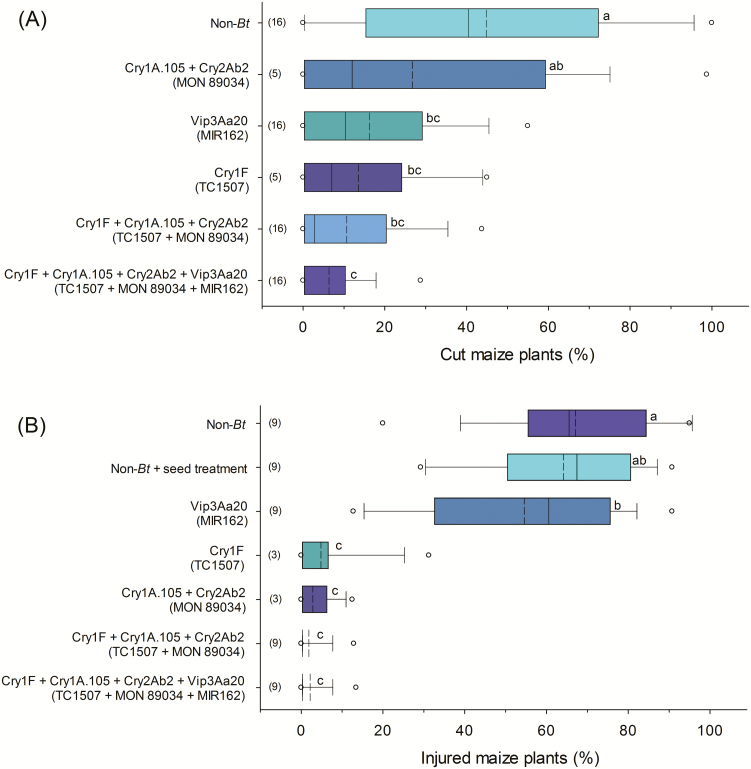
Mortality of Bt maize plants caused by (A) *Agrotis ipsilon* and (B) *Elasmopalpus lignosellus*, 15 and 21 d after artificial field infestation, respectively. The dashed line within a box represents the mean. The solid line within a box represents the median. The ‘o’ in the boxplots represents 95th percentiles. Boxplots followed by different letters were significantly different by Tukey’s test (α = 0.05). The vertical axis lists protein names and, in parentheses, the names of the transgenic events.

The percent of plants cut by *A. ipsilon*, 15 DAI, was significantly lower (*F*_5, 54_ = 18.94; *P* < 0.0001) in most Bt maize technologies compared to the non-Bt iso-hybrid, except in maize expressing Cry1A.105 + Cry2Ab2 (MON 89034) (Fig. 2A). The percent of plants cut in the non-Bt iso-hybrid was 45.2 ± 3.6, whereas in the Bt maize technologies, the highest level of cut plants was observed in maize with Cry1A.105 + Cry2Ab2 proteins (MON 89034) with 26.3 ± 7.1%. The percent of plants cut in this technology did not differ significantly from those in single events MIR162 (15.9 ± 2.2) and TC1507 (13.2 ± 3.4), or from Bt maize expressing Cry1F + Cry1A.105 + Cry2Ab2 proteins (TC1507 × MON 89034). The lowest percentage of plants cut by *A. ipsilon* (6.0 ± 1.2) was observed in the technology with the Bt pyramid Cry1F + Cry1A.105 + Cry2Ab2 + Vip3Aa20 (TC1507 × MON 89034 × MIR162), and was statistically similar to the percentage of cut plants on Bt maize with Cry1F (TC1507), Vip3Aa20 (MIR162), and Cry1F + Cry1A.105 + Cry2Ab2 (TC1507 × MON 89034) (Fig. 2A).

Bt maize technologies expressing Cry1F (TC1507) and Cry1A.105 + Cry2Ab2 (MON 89034) and the combination of both, as well as the maize technology with Cry1F + Cry1A.105 + Cry2Ab2 + Vip3Aa20 (TC1507 × MON 89034 × MIR162), showed less than 5% of plants injured (dead heart and tillering) caused by *E. lignosellus*. The injury observed in these technologies was significantly lower compared to the injury observed in the maize technology with single event MIR162 and in the non-Bt iso-hybrid with and without a seed treatment (*F*_6, 36.3_ = 76.14; *P* < 0.0001) (Fig. 2B). Although the single event MIR162 did not reduce plant injury caused by *E. lignosellus* to the same extent as the other Bt maize technologies, the percent of injured plants in this single event (54.1 ± 4.1) was significantly lower compared to the non-Bt iso-hybrid without the seed treatment (66.5 ± 3.3), and was statistically similar to the non-Bt iso-hybrid with a seed treatment (63.6 ± 3.3).

## Discussion

Results from 3 yrs of field evaluations across four maize-producing states in Brazil indicate that the pyramided Bt technology Cry1F + Cry1A.105 + Cry2Ab2 + Vip3Aa20 (events TC1507 × MON 89034 × MIR162) provided higher and broader protection from feeding damage by a lepidopteran pest complex composed of *H. zea*, *D. saccharalis, E. lignosellus* and *A. ipsilon* compared to the other tested commercial Bt maize technologies.

Cry1F + Cry1A.105 + Cry2Ab2 + Vip3Aa20 provided similar protection for *H. zea* kernel feeding, compared with Vip3Aa20 alone, while Bt maize technologies with single events TC1507 (Cry1F protein) and MON 89034 (Cry1A.105 + Cry2Ab2 proteins) did not significantly reduce kernel-feeding injury by *H. zea* in this study. The results on *H. zea* are consistent with previous studies in which sweet corn or maize containing Vip3Aa20 alone or combined with other transgenic proteins provided superior control compared with other Bt proteins based on trials in the United States (Buntin 2008, Burkness et al. 2010, Siebert et al. 2012, Reay-Jones and Reisig 2014, Reisig et al. 2015, Yang et al. 2015, Dively et al. 2016, Reay-Jones et al. 2016). The combination of events TC1507 and MON 89034 in the pyramid Cry1F + Cry1A.105 + Cry2Ab2 offered a slight improvement in protection from the non-Bt, albeit non-significant from that of their separate events. Therefore, the excellent levels of control of kernel feeding in the pyramided technology Cry1F + Cry1A.105 + Cry2Ab2 + Vip3Aa20 (TC1507 × MON 89034 × MIR162) were likely due to the high activity of Vip3Aa20 on this pest compared to the other Bt proteins.

All maize hybrids evaluated in our study and containing a single or pyramided Bt proteins were highly effective in reducing tunneling injury caused by *D. saccharalis*. These results are consistent with those reported by Wangila et al. (2013) who reported that SmartStax transgenic maize (SmartStax multi-event technology developed by Dow AgroSciences LLC, Indianapolis, IN and Monsanto Co., St. Louis, MO), which expresses Cry1F, Cry1A.105, and Cry2Ab2, was effective for controlling *D. saccharalis* and for protecting plant injury from this insect. Pyramided Bt maize hybrids containing Cry1A.105 + Cry2Ab2 proteins or SmartStax multi-event technology were effective against *D. saccharalis* in greenhouse studies with artificial infestations of three pest genotypes (Cry1Ab-susceptible, Cry1Ab-resistant, and heterozygous) of *D. saccharalis* (Wangila et al. 2012). Wu et al. (2009) demonstrating that Cry1A.105 and Cry2Ab2 proteins have activity against Cry1Ab-susceptible and Cry1Ab resistant *D. saccharalis* in laboratory studies, with larvae exhibiting greater sensitivity to Cry1A.105. Furthermore, field studies evaluating maize hybrids containing both Cry1A.105 and Cry2Ab2 proteins provided complete control of Cry1Ab-susceptible and resistant genotypes of *D. saccharalis* (Ghimire et al. 2011). Bt maize hybrids containing Cry1F, Cry1A.105 + Cry2Ab2 or Cry1F + Cry1A.105 + Cry2Ab2 demonstrated to be effective against *D. saccharalis* (Siebert et al. 2012; Okumura et al. 2013). Waquil et al. (2013) also reported that Cry1A.105 + Cry2Ab2 maize was effective in controlling *D. saccharalis* in Brazil. According to Bernardi et al. (2015), Bt maize expressing Vip3Aa20 protein provided 100% mortality on *D. saccharalis* in laboratory trials and had low corn stalk damage in field studies. Our studies also agree with Okumura et al. (2013) that reported field maize hybrid expressing Vip3Aa had no *D. saccharalis* injury and 100% of control of this pest.

Bt maize hybrid technologies with transgenic events TC1507, MON 89034, TC1507 × MON 89034, and TC1507 × MON 89034 × MIR162 protected plants against dead heart and tillering injury caused by *E. lignosellus*. However, Bt maize containing only the Vip3Aa20 protein or non-Bt maize with seed-applied thiamethoxam at 42 g a.i./60.000 seeds, did not provide consistent protection against this pest. Our results are consistent with those by Siebert et al. (2012) from the southeast and mid-south United States in which plant mortality induced from *E. lignosellus* infestations was significantly less and number of larvae was significantly lower for Bt maize hybrids containing Cry1F, Cry1A.105 + Cry2Ab2, Cry1F + Cry1A.105 + Cry2Ab2 as compared with a non-Bt maize hybrid. Bt field maize hybrids that contain Cry1F protein provided high levels of efficacy against *E. lignosellus* (Vilella et al. 2002, Reisig et al. 2015). Injury data from *E lignosellus* are consistent with findings of previous reports in which Cry1F, and Cry1A.105 + Cry2Ab2 proteins provide effective management of *E. lignosellus*, while Vip3Aa single protein did not protect maize plants against this pest in field experiments conducted in Lucas do Rio Verde, in Mato Grosso State, Brazil (Okumura et al. 2013).

Our results on the efficacy against *A. ipsilon* resemble those by Rule et al. (2014) who reported the percent plant stand reduction in seedling stage maize was significantly lower for Bt hybrids expressing Cry1F and the pyramid of Cry1F + Cry1A.105 + Cry2Ab2 proteins compared with the non-Bt and Cry1A.105 + Cry2Ab2 hybrids. Kullik et al. (2011) reported greenhouse and field studies in Ontario, Canada, in which Cry1F-expressing maize hybrids consistently had the highest plant population densities after naturally occurring black cutworm infestation. The development and adoption of locally-adapted integrated pest management with focus on resistance management and on best management practices is essential to agricultural sustainability. The use of insecticide at burndown, when applicable, insecticide seed treatment, crop and pest scouting, complementary insecticide applications based on resistance management thresholds, weed control, and crop rotation (rotating mode of actions or host to non-hosts, when possible) are critical to the sustainability of biotechnologies. Bernardi et al. (2016) cited the use of seed treatments as a tool compatible with Bt crop technology to bring additional control of seedling pests and minimize the need of foliar insecticides during developmental stages of the crop. The use of seed treatments, within the context of an Insect Resistance Management (IRM) program could help the establishment of non-Bt plants in the refuge area, maintaining the first criteria for yield potential in refuge areas (initial stand), as well as serving as an additional mode of action in Bt crops in controlling target pests of Bt crops during the period when the seed treatment is effective. Seed treatments combined with Bt technology are simple for the grower to deploy, and would be deployed widely across the landscape, thus enhance their effectiveness in IRM programs. Seed treatments should be evaluated closely with new pyramided Bt technologies to assess their potential contribution and value as an additional tool to further protect from crop injury caused by Bt target and non-target pests, and to enhance IRM strategies on Bt crops.

The rapid evolution of insect resistance to Bt transgenic traits is a high threat and a reality under Brazilian and South American conditions. Recent reports of resistance to Cry1F by *S. frugiperda* in Brazil and Argentina (Farias et al. 2014, Chandrasena et al. 2017), *D. saccharalis* to Cry1F and Cry1A.105 in Argentina (Grimi et al. 2018, Signorini et al. 2018) and multiple Bt trait resistance reports from other lepidopteran pest species across the world (Carrière et al. 2016a) are evidence of the pests’ inherent abilities to develop resistance to Bt crops. Bt crop pyramids have often been cited as one of several tools to delay resistance to Bt crops (Carrière et al. 2015, 2016a,b). However, the use of crops with pyramided traits must be supported by the continued use of proper non-Bt refuges near Bt crops (Carrière et al. 2016b). Three years of field efficacy experiments across important commercial maize-producing areas in Brazil demonstrated the high efficacy of pyramided Bt maize Cry1F + Cry1A.105 + Cry2Ab2 + Vip3Aa20 (events TC1507 × MON 89034 × MIR162) to control feeding injury caused by *H. zea, D. saccharalis, A. ipsilon*, and *E. lignosellus*. The sustainability of this new pyramided Bt maize, as with any other Bt technology, depends largely on the implementation of appropriate non-Bt refuge areas, the application of locally recommended best management practices and implementation of a comprehensive communication and training program for Bt crop management (Signorini et al. 2018) to ensure extended economic and environmental benefits to the Brazilian agriculture.
